# Protocols of a diagnostic study and a randomized controlled non-inferiority trial comparing televisits vs standard in-person outpatient visits for narcolepsy diagnosis and care: TElemedicine for NARcolepsy (TENAR**)**

**DOI:** 10.1186/s12883-020-01762-9

**Published:** 2020-05-11

**Authors:** Francesca Ingravallo, Luca Vignatelli, Uberto Pagotto, Stefano Vandi, Monica Moresco, Anastasia Mangiaruga, Claudia Oriolo, Corrado Zenesini, Fabio Pizza, Giuseppe Plazzi

**Affiliations:** 1 Department of Medical and Surgical Sciences (DIMEC), University of Bologna, Bologna, Italy; 2 IRCCS Istituto delle Scienze Neurologiche di Bologna, Via Altura 3, Bologna, Italy; 3 Department of Biomedical and Neuromotor Sciences (DIBINEM), University of Bologna, Bologna, Italy

**Keywords:** Telemedicine, Televisit, Diagnosis, Management, Care, Sleep disorders, Narcolepsy, Sleepiness, Quality of life

## Abstract

**Background:**

Narcolepsy is a rare chronic sleep disorder that typically begins in youth. Excessive daytime sleepiness is the main disabling symptom, but the disease is often associated with severe endocrine-metabolic and psychosocial issues, worsened by a long diagnostic delay, requiring a multidisciplinary approach. The scarcity of reference Sleep Centres forces the patient and family to travel for seeking medical consultations, increasing the economic and psychosocial burden of the disease. Growing evidence suggests that Telemedicine may facilitate patient access to sleep consultations and its non-inferiority in terms of patient satisfaction, adherence to treatment, and symptom improvement for sleep disorders. However, Telemedicine clinical and economic benefits for patients with narcolepsy are still unknown.

**Methods:**

TENAR is a two-part project, including: 1. a cross-sectional study (involving 250 children and adults with suspected narcolepsy) evaluating the accuracy of Teletriage (i.e., a synchronous live interactive sleep assessment through a Televisit) for narcolepsy diagnosis compared to the reference standard; and 2. a two-arm, parallel, open randomized controlled trial (RCT) to demonstrate the non-inferiority of the multidisciplinary care of narcolepsy through Televisits versus standard care. In this RCT, 202 adolescents (> 14 y.o.) and adults with narcolepsy will be randomly allocated (1:1 ratio) either to Televisits via videoconference or to standard in-person outpatient follow-up visits (control arm). The primary outcome is sleepiness control (according to the Epworth Sleepiness Scale). Secondary outcomes are other symptoms control, compliance with treatment, metabolic control, quality of life, feasibility, patient and family satisfaction with care, safety, and disease-related costs. At baseline and at 12 months, patients will undergo neurologic, metabolic, and psychosocial assessments and we will measure primary and secondary outcomes. Primary outcomes will be also measured at 6 months (remotely or in person, according to the arm).

**Discussion:**

TENAR project will assess, for the first time, the feasibility, accuracy, efficacy and safety of Telemedicine procedures applied to the diagnosis and the multidisciplinary care of children and adults with narcolepsy. The study may be a model for the remote management of other rare disorders, offering care access for patients living in areas lacking medical centres with specific expertise.

**Trial registration:**

Number of the Tele-multidisciplinary care study NCT04316286. Registered 20 March 2020.

## Background

Narcolepsy is a rare chronic disorder of hypersomnolence [1] with excessive daytime sleepiness (EDS) as the main disabling symptom. The estimated prevalence in the general population is of 0.02–0.067% [2]. Narcolepsy onset occurs between childhood and young adulthood [3]. However, patients often experience an extended diagnostic delay (14 years in Europe) [4] that may be associated with misdiagnosis and inappropriate resource utilization, increasing the psycho-social impact of the disease [3, 5]. Quality of life and social activities are profoundly impaired in both adults and children [6, 7], and patients need frequent and timely counselling to adjust behavioural and pharmacological treatments. High direct and indirect disease-related costs are reported [8–10]. Metabolic (obesity, diabetes, precocious puberty) and psychiatric comorbidities (mood, anxiety, and eating disorders) are additional common features in narcolepsy [11], and require a multidisciplinary approach. To our knowledge, the ‘Centro Narcolessia’ (CN) of Bologna, located in the Emilia-Romagna Region in the north of Italy, is the only Italian centre offering a multidisciplinary management of narcolepsy (covering sleep, metabolic, psychological, and psychosocial aspects for both paediatric and adult patients), avoiding multiple referrals. The CN is the first Italian centre in terms of number of assisted patients, 71% of whom come from outside the Emilia-Romagna Region (the most distant one comes from Pantelleria, an island 1553 km far from the CN). Unmet patients’ needs at regional level force them to undertake long journeys to seek for consultations, with consequent delayed access to diagnosis and care and high direct and indirect disease-related costs, possibly worsening the control of disease symptoms and the prevention of drug side effects. Indeed, according to the Authors’ clinical practice experience, patients may refrain from scheduled outpatient visits due to high costs to reach the Sleep centre.

In this regard, Telemedicine (TM) is one of the most promising approaches. TM is the “practice of medicine using electronic communications, information technology, or other means between a licensee in one location and a patient in another location” [12]. The American Academy of Sleep Medicine supports TM as a means of advancing patient health by improving access to the expertise of qualified Sleep Medicine specialists [13]. TM includes Televisits (TVs) via videoconference, a synchronous live interactive TM visit, in which “patients and providers are separated by distance but interact in real-time utilizing videoconferencing as the core technology” [13].

Several studies about the impact and outcomes of Sleep TM investigated various TM applications (e.g., TVs, home sleep test devices or telemetric titration methods, etc.) for the diagnosis, monitoring, treatment, education and follow-up of obstructive sleep apnoea [14–17]. Despite the scarceness of randomized controlled trials (RCT), the available studies suggest the non inferiority of TM vs in-person care in terms of patient satisfaction, adherence to treatment, and symptomatic improvement [17]. Moreover, some studies confirm that TM has the potential to facilitate patient access to sleep consultations [18–21]. However, clinical and economic benefits of Sleep TV procedures is still uncertain [22], and its impact, with the exception of stroke, has been rarely explored in the neurology field [23]. In particular, the potential of this innovative approach for the diagnosis and management of narcolepsy is still unknown. Robust randomized trials are needed to understand the impact of Sleep TM on patient outcomes, and health-care system accessibility [17].

The TENAR (TElemedicine for NARcolepsy) project will assess for the first time Sleep TM procedures for children and adults with narcolepsy. In particular, the project will assess the feasibility, accuracy, efficacy and safety of TVs applied to the diagnostic process and the multidisciplinary care of narcolepsy. The TENAR project combines the patient-oriented outcomes research approach with the theoretical approach of “move the healthcare where it really needs” [24] through TM and mobile internet devices.

## Aims

TENAR is a two-part project aiming at: 1. assessing the accuracy of TV for the diagnosis of narcolepsy (*Tele-triage study*) in children and adults with suspected narcolepsy; and 2. demonstrating the non-inferiority for EDS control of the multidisciplinary management through TVs vs standard in-person outpatient follow-up visits, while monitoring patient-centred outcomes (*Tele-multidisciplinary care – TMC - study*). The TENAR project is endorsed by the Italian Narcolepsy and Hypersomnias association (AIN onlus, www.narcolessia.org).

## Methods/design

TENAR is a monocentric project designed by the CN (‘Centro Narcolessia’ - Narcolepsy Centre), part of IRCCS Istituto delle Scienze Neurologiche di Bologna, an academic hospital, in the city of Bologna, Italy. The coordination of the project, data collection, management, analysis, and interpretation of data, writing of the reports will be performed by the CN. All Authors will have access to the final dataset.

### Tele-triage study

#### Study design and outcomes

The Tele-triage study aims at assessing the diagnostic accuracy of a sleep medicine assessment through a TV of patients with suspected narcolepsy, by means of a cross-sectional design. The index text will be the diagnostic judgment after a TV by a sleep specialist according to three levels: “probable narcolepsy”, “possible narcolepsy” or “excluded narcolepsy”. The reference standard will be the final diagnosis (“confirmed narcolepsy” or “excluded narcolepsy”), established after a standard work-up for the diagnosis of narcolepsy and other sleep disorders with EDS complaint according to the International Classification of Sleep Disorders (ICSD-3) criteria [1] (i.e. symptoms and objective in laboratory assessment, eventually including cerebrospinal fluid orexin level measurement). A physician blinded to the initial diagnostic suspicion will establish the final diagnosis on the basis of patients records, including test results. The primary outcome is the diagnostic accuracy (sensitivity, specificity); the secondary outcome is the inter-observer agreement between sleep experts on diagnostic orientation (according to 3 levels: “probable narcolepsy”, “possible narcolepsy”, or “narcolepsy excluded”) after in-person or TV assessment respectively.

#### Participants, setting and procedures

Children (any age) and adults admitted to CN outpatient clinic of the IRCCS Istituto delle Scienze Neurologiche di Bologna, Italy, for suspected narcolepsy will be invited to participate. After informed consent procedure, participants will undergo a sleep assessment through in-person office visit first or TV first, by two independent sleep experts. Both the type of visit and the examiner will be assigned randomly. During TV, patients will use a tablet (in a dedicated CN room) and the sleep expert an in-office PC (in a different room). In case of children, a parent/caregiver will be present at both assessments. The sleep assessment for suspected narcolepsy consists of a reliable semi-structured interview on narcolepsy symptoms [25].

At the end of the assessment, each examiner will express his/her diagnostic orientation (“probable”, “possible” or “excluded” diagnosis of narcolepsy). In the following months, all participants will undergo the reference standard work-up according to clinical practice.

#### Sample size and statistical considerations

Two hundred and fifty patients are necessary to prove the desired sensitivity of 94% (CI lower limit) of TV, keeping CI lower limit of specificity at 73%, expecting 40% prevalence of narcolepsy at the CN outpatient clinic referral. Interobserver reliability will be calculated by kappa statistics, which is the ratio of the observed agreement beyond chance to the potential agreement, according to the formula proposed by Fleiss [26]. Kappa values will be interpreted according to conventional groups (0.0–0.20 = slight agreement, 0.21–0.40 = fair, 0.41–0.60 = moderate, 0.61–0.80 = substantial, 0.81–1.00 = almost perfect) [27].

### Tele-multidisciplinary care (TMC) study

#### Study design and outcomes

**Fig. 1:**
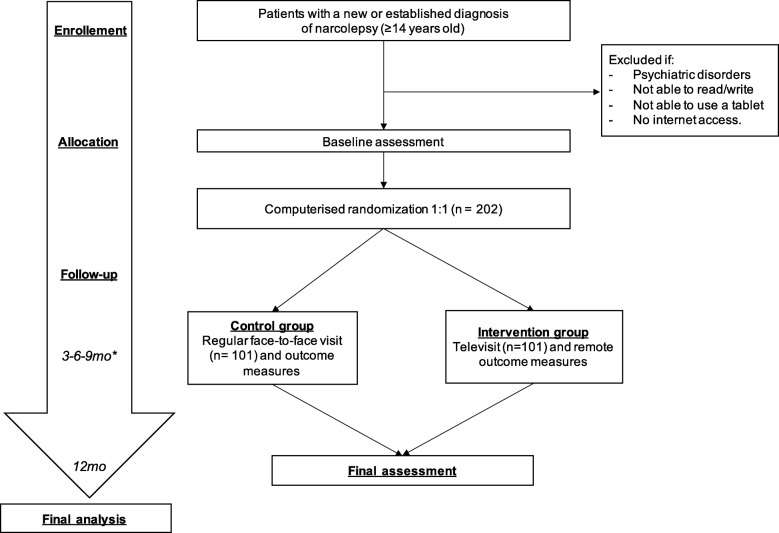
Flowchart of the randomized controlled trial

The TMC study is a 12-month, two-arm, parallel, open RCT aiming to demonstrate the non-inferiority for EDS control of the multidisciplinary care of narcolepsy through TVs versus standard in-person follow-up care (Fig. 1). Primary outcome is EDS control (according to the Epworth Sleepiness Scale, ESS [28]). Secondary outcomes are the following: other symptoms control, compliance with treatment, metabolic control, quality of life, feasibility, patient and family satisfaction with care, safety, and disease-related costs. Primary and secondary outcomes, and related measures and timepoints for data collection are summarized in Table 1.

**Table 1:** Primary and secondary outcomes, measures and timepoints

Outcomes	Measures	Timepoints (months)
Primary		
EDS	ESS (adults) [28] ESS-CHAD [32] (adolescents)	0, 3, 6, 9, 12 incident patients 0, 6, 12 prevalent patients
Secondary		
Other symptoms	Cataplexy diary, NSS [33], BDI [34], STAI [35] (adults) CDI2 [36], MASC [37] (adolescents) Global Impression Scale [38] (physicians)	0, 3, 6, 9, 12 incident patients 0, 6, 12 prevalent patients
Compliance to treatment	Clinical consultation	0, 3, 6, 9, 12 incident patients 0, 6, 12 prevalent patients
Weight	Standard weight scale provided to each patient	0, 3, 6, 9, 12 incident patients 0, 6, 12 prevalent patients
Lipidic/glycaemic control*	Laboratory tests	0, 12
Caloric intake	Food diary	0, 12 [0, 3, 6, 9, 12 patients with metabolic problems]
Physical activity	Short-IPAQ [29]	0, 12 [0, 3, 6, 9, 12 patients with metabolic problems]
Satisfaction index	CoTenar (patients and caregiver)**	6, 12
Quality of life	SF-36 (adults) [39] PedsQL [40] (adolescents)	0, 6, 12
Number (%) of full dropouts		At the time of censorship
Number (%) of partial dropouts (patients changing procedure)		At the time of arm change
Adverse drug reactions	Clinical consultation	0, 3, 6, 9, 12 incident patients 0, 6, 12 prevalent patients
Costs and accidents	Interview (patients and caregivers)	0, 6, 12
Other feasibility indexes	Failure system report (physicians and patients)	After each TV

*Glucose, insulin, HbA1c, triglycerides, total, LDL and HDL cholesterol, TSH, glycaemia, insulin, glycated haemoglobin, HDL, triglycerides, total, LDL, uricemia, TSH, FT4, FT3, complete blood count, AST, ALT, gamma-GT, alkaline phosphatase, creatinine. ** Adapted from CoSM-S [41]

#### Population, intervention and procedures

Adolescents (≥14 years old) and adults with a diagnosis of narcolepsy according to ICSD-3 [1] criteria soon after the diagnosis (“incident”) or with established diagnosis (“prevalent” subjects) in charge at the CN of the IRCCS Istituto delle Scienze Neurologiche di Bologna, Italy, and able to provide consent are eligible. Exclusion criteria are the following: major psychiatric disorders; inability to read, write, or using a tablet; and no Internet access.

Patients who provide informed consent will undergo the following baseline in-person assessment by different experts through standardized tools (see Table 1):

- sleep assessment;
- endocrine/metabolic assessment, with measurement of weight, height, waist circumference, blood pressure, heart rate; and
- a counselling session on the psychosocial impact of narcolepsy.

Patients will also be requested to complete a physical activity questionnaire [29], and to provide a 24-h food diary. In addition, a blood sample will be collected for a metabolic (lipid and glucose profile, and thyroid function) evaluation.

After baseline procedures, patients will be consecutively randomly allocated in 1:1 ratio either to TMC via videoconference or to standard in-person follow-up in-office visits (control arm). The randomization list (stratified for three groups: children, adults with incident condition, adults with prevalent condition) and allocation will be generated by an automatic web-based system after inclusion.

The TMC will consist of scheduled TVs through a tablet and remote data collection through an ad hoc designed electronic case report form (e-CRF). A videoconference service will be used. The TV will be performed aiming at assessing the patient’s condition, and his/her need/response to treatments. TVs will end with therapeutic prescriptions according to usual practice, until the next follow-up TV. A formal report of the sleep assessment through TV will be sent by mail to the patient. The standard in-person follow-up care consists in scheduled (3–6-9 months) usual in-office visits, performing the same assessments, data collection and therapeutic prescriptions according to usual practice. The scheduling of follow-up TVs or in-person visits (at 3–6-9 months) will be based on the patient’s condition and needs (see Table 1).

#### Sample size and statistical considerations

A total of 176 patients (88 per arm) are required to prove that the 95% lower limit of one-sided CI will be above the non-inferiority limit of − 1.5 ESS points difference (standard deviation of 4), with 80% of power. A previous RCT used a non-inferiority margin of 2 ESS points and 80% as minimal power to compare pitolisant versus modafinil in patients with narcolepsy [30]. As the TMC study will not test drugs, we decided to adopt a stricter margin (1.5 ESS points). The standard deviation value (±4) was based on the Italian validation study of ESS [28]. Considering 15% of dropouts, the sample was enlarged to 202 (101 per arm). Repeated measures ANOVA will be used to compare EDS between groups at baseline and after 1-yr of follow-up.

#### Safety assessments and monitoring

Patients may face difficulties in accepting TMC for the fear of less accurate assistance or for barriers in the use of mobile technology. We plan to cooperate with AIN for the recruitment and education (e.g., tutorial activities for the tablet use) in order to avoid inappropriate exclusion of patients with technology barriers. The patient, contacting a provider through the tablet, 3-h/week, may request on-call TVs. The same availability will be provided to patients included in the control arm.

With patient consent, the first TVs will be recorded in order to assess the experts’ fidelity to the protocol procedures. Recordings will be destroyed at the end of the assessment. CN will be in charge of monitoring.

#### Data entry, coding, security, and storage

All data will be entered electronically in an ad-hoc design web-based e-CRF. This will be done directly by the patient filling in the forms before each visit (e-questionnaires), or during the visits by the physicians (clinical assessment). The e-CRF is designed and validated for data security and storage according to international standards (US FDA: 21 CFR Part 11; EU GMP: Annex 11; ICH/GCP; VICH/GCP; GAMP 5; GDPR). Personal information about participants will be processed in accordance with the GDPR and national regulation on data protection.

### Ethical approval and informed consent procedures

The study will be conducted in accordance with Helsinki declaration. The Independent Ethics Committee of Area Vasta Emilia Centro (CE-AVEC) approved both the Tele-Triage study and the TMC study (number 18034-120_2018_sper and 18,033-121_2018_sper respectively). Signed informed consent form will be obtained by the sleep experts prior to recruitment. Consent for patients< 18 y.o. will be provided by their parents. Separate assent forms will be signed by minors (aged 12–17 and aged 14–17 for Tele-Triage study and TMC study respectively). Both participants and parents/guardians will be free to withdraw their consent for participation at any time. Participants may request to be informed about results of the study once the study is finished. The results of the TENAR project will be communicated during scientific meetings and AIN’s meetings, and through publication in peer-reviewed journals. We will not involve a professional writer.

## Discussion

Narcolepsy is a rare chronic disease with complex needs encompassing medical and psychosocial aspects. The TENAR project will provide, for the first time, evidence about the feasibility, accuracy, efficacy, and safety of TM procedures applied to the diagnosis and the multidisciplinary care of children and adults with narcolepsy. The project combines a multidisciplinary patient-centered approach to narcolepsy with the possibility of screening and following up patients sparsely living across the country by erasing distance-related barriers. We hypothesize that the Tele-triage and the TMC will guarantee accurate, efficient and safe screening and care of patients with narcolepsy.

First, we expect that the Tele-triage procedure will have a good diagnostic accuracy, namely a high sensitivity in capturing possible patients with narcolepsy. This procedure may ease patient access to highly qualified Sleep centres, reducing the diagnostic gap and its detrimental consequences. This is paramount for people suffering from narcolepsy, especially for the youngest ones. Delayed diagnosis, misdiagnoses, and multiple referrals before receiving a proper diagnosis are frequent in narcolepsy [31] and have negative consequences for untreated patients, their family and the healthcare system. Indeed, the diagnostic gap aggravates the psychosocial and economic burden of the disease [3, 31], while a prompt diagnosis of narcolepsy at a young age seems to reduce the future socio-economic burden of the disease [5].

Second, we expect that the TMC will improve patient-centred outcomes without worsening EDS control. TMC guarantees a face-to-face interaction between patients and physicians working in qualified third level centres care, overcoming the geographical barriers.

If the study results will be in line with our hypotheses, the TENAR study procedures will potentially improve access to a timely diagnosis and care for patients with narcolepsy in Italy and other countries. Given the Directive 2011/24/EU on the application of patients’ rights in cross-border healthcare (http://data.europa.eu/eli/dir/2011/24/oj), and the European Reference Networks for rare diseases (http://www.ern-rnd.eu/), the TENAR project approach may also be a model for the management of other rare disorders, and will offer solutions for remote care of patients residing in areas lacking of specific expertise.

## Data Availability

This manuscript does not contain any data. At the end of the study, datasets and materials will be available from the corresponding Author on reasonable request.

## Abbreviations

BDI: Beck Depression Inventory
CDI2: Children Depression Inventory-2
EDS: Excessive daytime sleepiness
ESS: Epworth Sleepiness Scale
ESS-CHAD: Epworth Sleepiness Scale for Children & Adolescents
ICSD-3: International Classification of Sleep Disorders third edition
MASC: Multidimensional Anxiety Scale for Children
NSS: Narcolepsy Severity Scale
PedsQL: Paediatric Quality of Life Inventory
SF-36: Short Form-36 health survey
Short-IPAQ: Short International Phisycal Activity Questionnaire
STAI: State-Trait Anxiety Inventory
TM: Telemedicine
TV: Televisit
VC: Video consultation
